# Ultrasound-Guided Vascular Access Is an Important Tool to Prevent Catastrophes: Transinferior Epigastric Artery Cardiac Catheterization

**DOI:** 10.1155/2018/2041643

**Published:** 2018-11-08

**Authors:** Ahmed Amro, Kanaan Mansoor, Mohammad Amro, Amal Sobeih, Rameez Sayyed

**Affiliations:** ^1^Department of Cardiovascular Services, Marshall University Joan C. Edwards School of Medicine, Huntington, WV, USA; ^2^Department of Internal Medicine, Marshall University Joan C. Edwards School of Medicine, Huntington, WV, USA; ^3^School of medicine, MUST-Misr University for Science & Technology, Cairo, Egypt; ^4^School of medicine, Al-Najah University, Nablus, State of Palestine

## Abstract

We report a case of cardiac catheterization that was done entirely by accidentally accessing the inferior epigastric artery (IEA) through an unintentional puncture of the U-shaped portion of the inferior epigastric artery. Luckily the patient did not have any trauma to the IEA and was d/c home with no complications. A 48-year-old female with history of hypertension and CAD S/P left circumflex stent many years ago who presented to our facility with persistent crescendo angina for which decision was made to proceed with LHC. The cardiac catheterization showed no significant CAD with patent stent so it was decided that there is no further intervention needed. Femoral angiogram was done and showed that the stick was high and the tip of the sheath was about to come out of the CFA; at the same time, it came into our minds that the sheath could be passing through the IEA by sticking the U portion of the IEA, but due to the high risk, an immediate access was obtained through the contralateral groin then a balloon over the wire was passed beyond the original sheath tip, then the sheath was slowly pulled back while contrast was injected. Angiogram showed that the sheath was inserted through the U-shaped portion of the IEA. *Conclusion*. Ultrasound guidance should be the first-line standard for arterial access in any cardiac catheterization procedure. US is a proven tool that can increase success and decrease complications in a wide variety of vascular access procedures.

## 1. Background

A major cause of morbidity and mortality during cardiac catheterization and percutaneous intervention is vascular access site complications [1]. Although the transradial approach has gained favor over the transfemoral as the access of choice due to fewer complications, transfemoral access is still necessary for procedures that require large bore access such as structural procedures, high-risk coronary interventions, and mechanical circulatory support procedures [2]. In the recent past research, much progress has been made in reducing the femoral access site complications such as arteriovenous fistula formation, hematoma development, an occurrence of arterial dissection, and retroperitoneal hemorrhages [3]. Though femoral access site complications have significantly reduced, the complication rate still looms around 3.7 to 4% [4, 5]. One such complication is puncture of the inferior epigastric artery which in the past has been reported to have led to catastrophic complications such as perforation, laceration, and dissection which lead to retroperitoneal hemorrhages [6]. Ultrasound is a modality that is not limited to formal radiological procedures anymore, but it is increasingly being utilized around the hospitals for various procedures [3]. Point of care ultrasounds have improved outcomes of multiple interventions, and hence, it would only be fair to employ this technique to avoid complications in the cardiac catheterization lab. We report a case in which entire cardiac catheterization was performed by accidentally accessing the inferior epigastric artery (IEA) through an unintentional puncture of the U-shaped portion of the inferior epigastric artery.

## 2. Case Presentation

**Figure 1:**
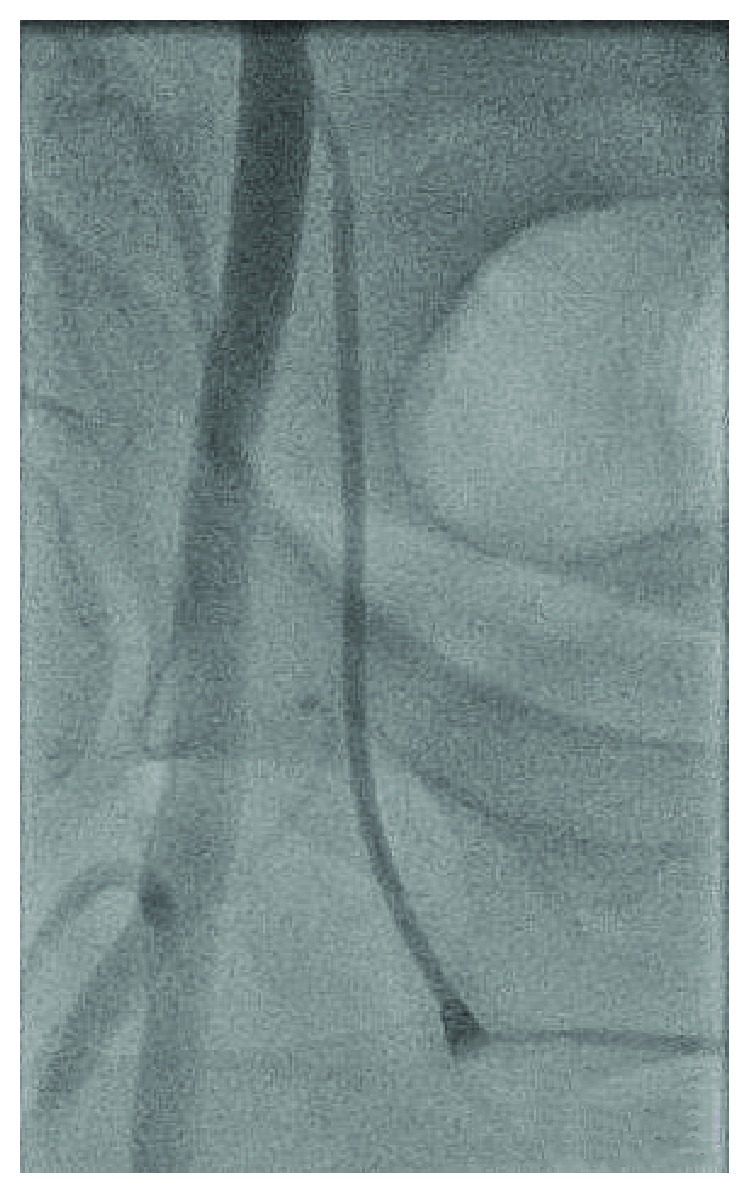
Questionable high stick with the sheath tip about to come out of the CFA.

**Figure 2:**
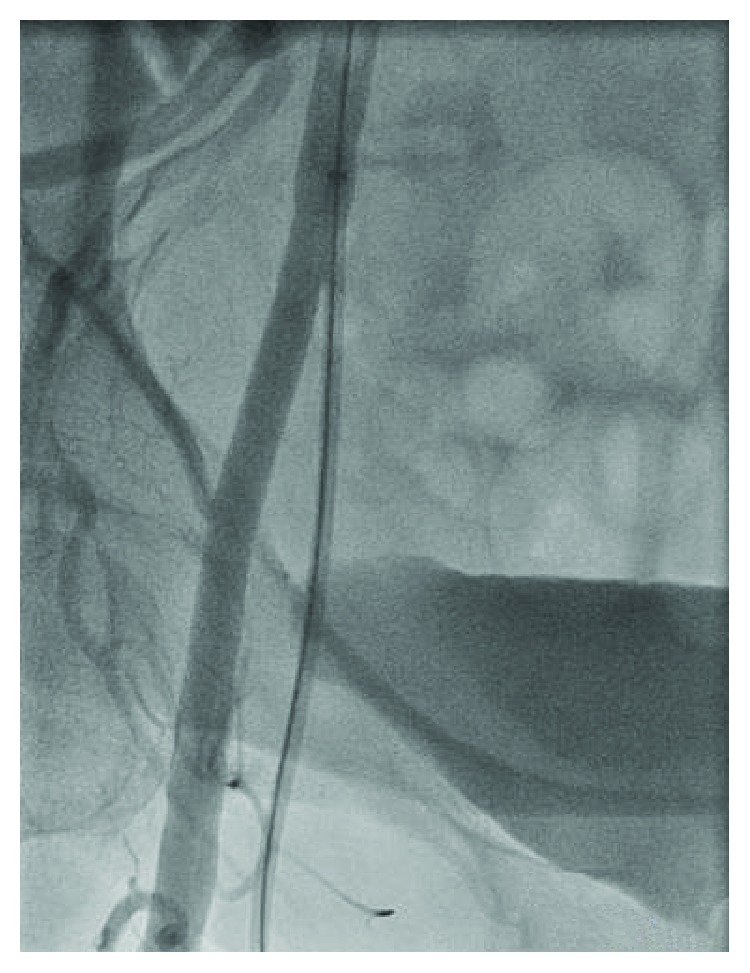
A wire passed through the sheath in order to secure access.

**Figure 3:**
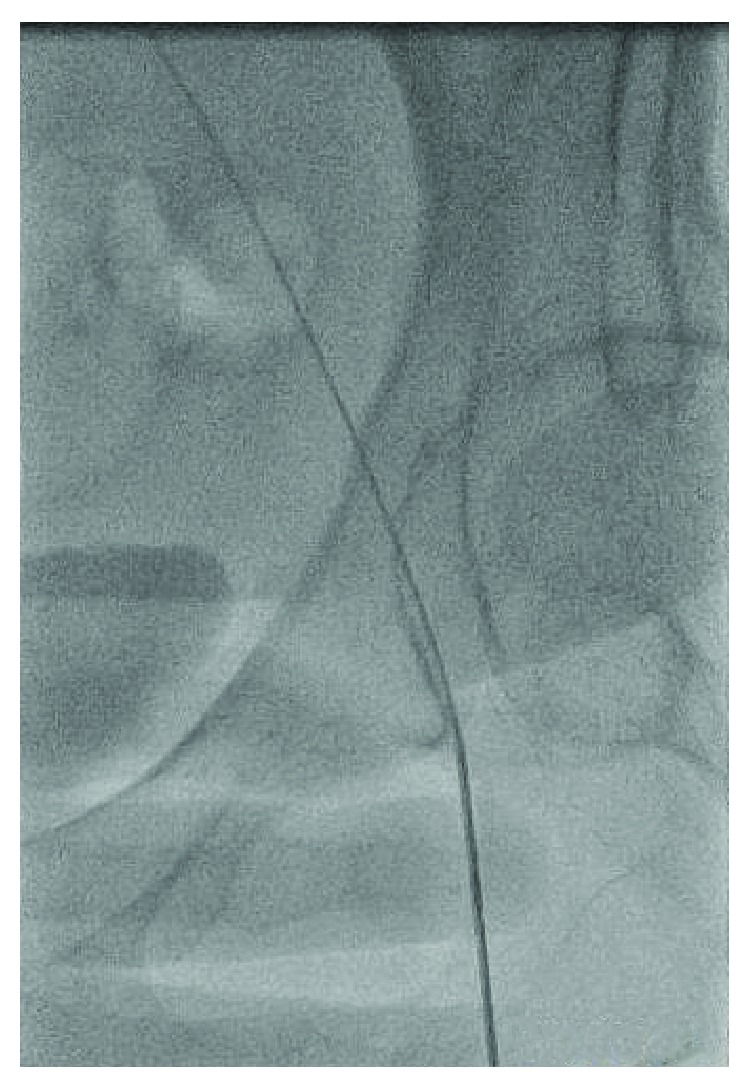
Access obtained from contralateral femoral artery.

**Figure 4:**
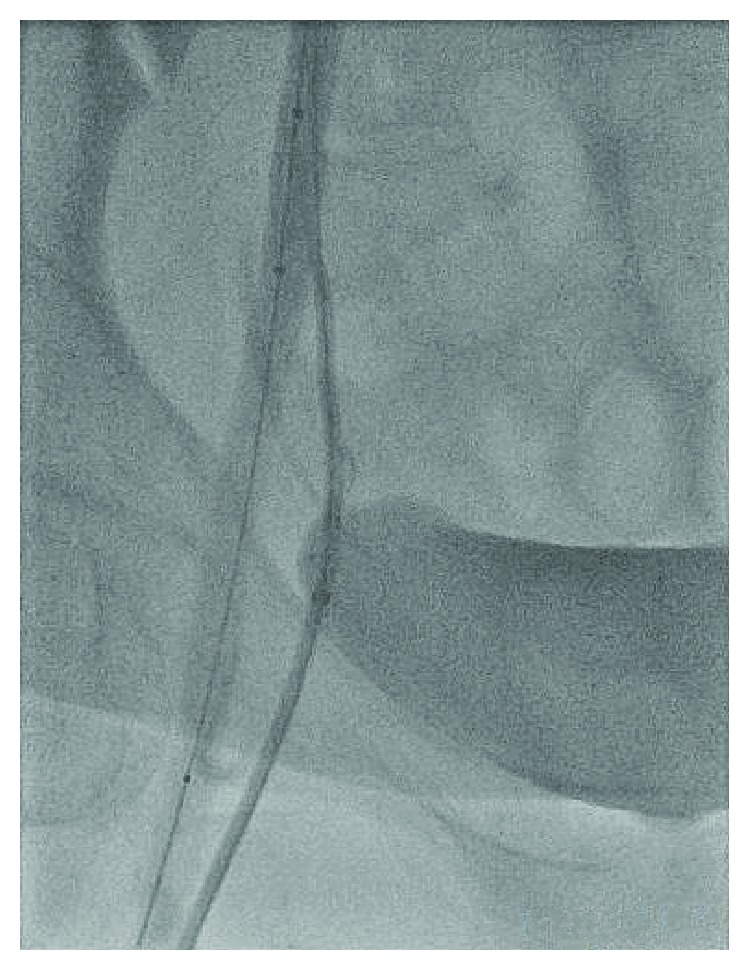
A balloon over the wire was passed beyond the original sheath tip.

**Figure 5:**
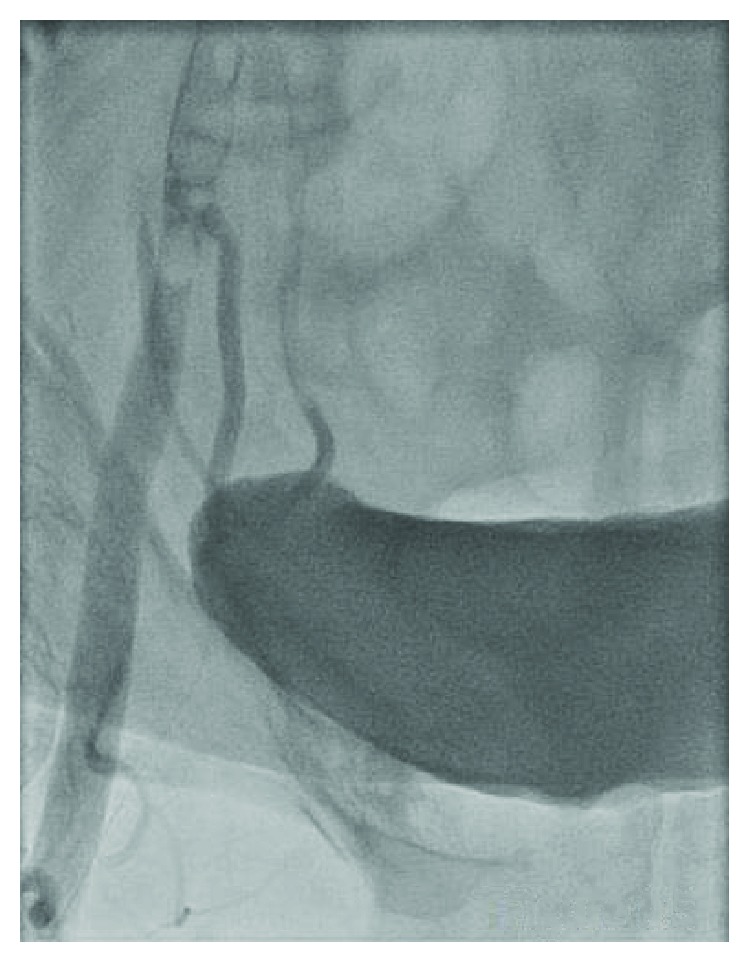
Angiogram showed that the sheath was inserted through the U-shaped portion of the IEA.

A 48-year-old female with a history of hypertension and CAD S/P left circumflex stent many years ago who presented to our facility with persistent crescendo angina for which decision was made to proceed with LHC. A micropuncture needle was used to obtain femoral access after fluoroscopy was used for anatomical localization of the CFA. A 6 F slender sheath was inserted and flushed. The cardiac catheterization showed no significant CAD with a patent stent, so it was decided that there is no further intervention needed. At the end of the procedure, it was suggested to use a closure device, so femoral angiogram was done at the end to assess the arteriotomy site which showed that the stick was high and the tip of the sheath was about to come out of the CFA (Figure 1); at the same time, it came into our minds that the sheath could be passing through the IEA by sticking the U portion of the IEA, but due to the high risk, a wire was passed through the sheath in order to secure access (Figure 2). Immediate access was obtained through the contralateral groin (Figure 3) then a balloon over the wire was passed beyond the original sheath tip (Figure 4), and then the sheath was slowly pulled back while contrast was injected. Angiogram showed that the sheath was inserted through the U-shaped portion of the IEA (Figure 5) and that the IEA had no dissection nor laceration. In the end, a closure device (Mynx) was applied to the access site and hemostasis was achieved. The patient was followed in the hospital and discharged home with no complications. The patient was seen in the clinic with no complications.

## 3. Discussion

Conventionally, femoral access is sought by the palpation of arterial pulses and identification of the anatomical landmarks by fluoroscopy. As per new recommendations, four techniques should be utilized in a combination to achieve access to the femoral artery. These four techniques are (a) fluoroscopy, (b) ultrasound, (c) micropuncture access, and (d) femoral angiography [2].

In our case, we relied on fluoroscopy alone in achieving femoral access, which led to cardiac catheterization that was done entirely through the inferior epigastric artery, through unintentional puncture of the U-shaped portion of the inferior epigastric artery (IEA). The origin of the IEA can vary. In 76% of cases, the origin of the IEA is from the external iliac artery (EIA) above the inguinal ligament. In 12% of cases, IEA arises from the behind of inguinal ligament, while in 8% of the cases, it arises from the femoral artery and in 4% of cases, it can arise from a common trunk with an abnormal obturator artery [7]. IEA artery after its origin is found anterior to the CFA following a U-shaped course inferiorly prior to continuing superiorly; it is at this site that the IEA is vulnerable to unintentional punctures. Moza et al. in 2013 also reported this as the site of puncture [8], which is similar to our case.

In our case, it was the due diligence of the operator that a catastrophe was avoided. Cases have been reported where the patients were not fortunate enough. Sanchez and Helmy reported two cases of the perforation of the inferior epigastric artery during cardiac catheterization. Sanchez and Helmy were able to identify the perforation in case 1 after the contrast dye extravasated into the peritoneal cavity, but it inadvertently led to the delaying of PCI by a day. Case 2 as reported by Sanchez and Helmy was identified after the patient complained of abdominal pain and upon further investigation; it was revealed that the patient had developed a retroperitoneal hemorrhage with acute blood loss anemia for which the patient had to be catheterized again [6].

Injury to the IEA can lead to fatal consequences unless managed aptly, the traditional approach to manage IEA entails external compression, balloon tamponade [6], thrombin injection [9], coil, and gel foam embolization [10]. Moza et al. reported having used the Angio-Seal vascular closure device [8]. On the contrary, in our case, we used a Mynx closure device to attain homeostasis and the patient remained stable.

In recent years, it has been advocated that the real-time ultrasound prior to gaining access would be invaluable and would lead to fewer complications. Kalish et al. concluded from a retrospective study of the Vascular Study Group of New England database that ultrasound-guided (UG) vascular intervention was protective against hematoma (rate ratio (RR), 0.62; 95% CI 0.46 to 0.84), while the subgroup analysis revealed that UG intervention protected against hematoma in patients with age over 80 years, BMI of ≥30, and sheath size > 6 F [11]. Femoral Artery Access with Ultrasound Trial (FAUST) is the largest trial, of 1004 patients; it concluded that in comparison to fluoroscopy, ultrasound guidance did not have any difference in CFA cannulation, while the in-depth subgroup analysis revealed that UG had better first-pass success (86% vs. 43%, *p* < 0.0001), reduced number of attempts (1.3 vs. 3, *p* < 0.0001), reduced median time to access (136 s vs. 148 s, *p* = 0.003), reduced risk of venipuncture (2.4% vs. 15.8%, *p* < 0.0001), and reduced vascular complications (1.4% vs. 3.4%, *p* = 0.04) [12].

Point of care ultrasound despite being readily available across most of the hospitals in the United States of America has not been employed to gain access in vascular intervention. A small survey published by Soverow et al. in 2016 reported that 13.3% of the respondents utilized ultrasound, despite 90% respondents expressed that they would be comfortable to use them and 88% acknowledged that ultrasound was readily available [13].

## 4. Conclusion

Ultrasound guidance should be the first-line standard for arterial access in any cardiac catheterization procedure. US is a proven tool that can increase success and decrease complications in a wide variety of vascular access procedures.
